# Human Cell Atlas and cell-type authentication for regenerative medicine

**DOI:** 10.1038/s12276-020-0421-1

**Published:** 2020-09-15

**Authors:** Yulia Panina, Peter Karagiannis, Andreas Kurtz, Glyn N. Stacey, Wataru Fujibuchi

**Affiliations:** 1 Center for iPS Cell Research and Application (CiRA), Kyoto University, 53 Kawahara-cho, Shogoin, Sakyo-ku, Kyoto 606-8507 Japan; 2 BIH Center for Regenerative Therapies (BCRT), Charité—Universitätsmedizin Berlin, Augustenburger Platz 1, 13353 Berlin, Germany; 3 International Stem Cell Banking Initiative, 2 High Street, Barley, Herts SG88HZ UK; 4 National Stem Cell Resource Centre, Institute of Zoology, Chinese Academy of Sciences, 100190 Beijing, China; 5 Innovation Academy for Stem Cell and Regeneration, Chinese Academy of Sciences, 100101 Beijing, China

**Keywords:** Stem-cell research, Differentiation, Translational research, Classification and taxonomy, Stem-cell differentiation

## Abstract

In modern biology, the correct identification of cell types is required for the developmental study of tissues and organs and the production of functional cells for cell therapies and disease modeling. For decades, cell types have been defined on the basis of morphological and physiological markers and, more recently, immunological markers and molecular properties. Recent advances in single-cell RNA sequencing have opened new doors for the characterization of cells at the individual and spatiotemporal levels on the basis of their RNA profiles, vastly transforming our understanding of cell types. The objective of this review is to survey the current progress in the field of cell-type identification, starting with the Human Cell Atlas project, which aims to sequence every cell in the human body, to molecular marker databases for individual cell types and other sources that address cell-type identification for regenerative medicine based on cell data guidelines.

## Regenerative medicine: Precise cell identification will improve future therapies

The characterization of cells into more precise groups will improve the chances of selecting the best types of cells to use for regenerative medicine. Traditionally, cell types are defined by physiological and morphological markers and molecular properties. However, sequencing technologies are enabling researchers to classify cells into increasingly distinct subgroups. Wataru Fujibuchi at Kyoto University, Japan, and co-workers reviewed progress in cell-type identification to help guide regenerative medicine. They examined the insights gained from the Human Cell Atlas project, an international collaboration of over 1000 institutes across 71 countries. Participants in this project are sequencing cellular RNA and categorizing hundreds of thousands of individual cells, demonstrating that many assumptions about cells are too simplistic. Such data will inform regenerative therapies, for example, by selecting the “best” platelet-producing stem cells for blood donation.

## The Human Cell Atlas

The Human Cell Atlas (HCA) project aims to characterize all cells by single-cell analytical techniques, specifically single-cell RNA sequencing (scRNA-seq) and assay for transposase accessible chromatin sequencing (scATAC-seq) and to link this information to classic knowledge, namely, location, lineage, and type, as well as cell states, state transitions, and cell−cell interactions. The HCA was initiated to unify scRNA-seq data in a manner similar to the data compilation achieved in the Human Genome Project^1^. As of February 2020, the HCA project has participation from 1027 institutes in 71 countries, from which 81 laboratories have already posted scRNA-seq data for 34 organs and tissues, including the liver^2^, lung^3^, blood and immune systems^4^, plasma cells^5^, human cortex^6^, colon^7^, and retina^8^, with the total number of sequenced cells reaching 4.5 million. The HCA is an open science project, and its standard operating protocols (SOPs) are available on the Web. A standardized data analysis service called the Data Coordination Platform (DCP) is provided to the community. In the long term, the HCA project aims to define not only normal cells but also cells in specific disease states^1,9^.

The HCA project is generating tremendous amounts of data. For example, there are currently 148 members in the immune system group, which is conducting approximately 100 projects and is expected to include more than 1000 projects over time. One project^10^ has produced scRNA-seq data for 530,000 cells. The challenge for the HCA project is to create a universal classification system for all of these projects. Adding to this challenge are regional HCAs, such as HCA Asia, that focus on regional diseases. To assign cell types to all these cells, exhaustive analysis methods are needed. Once this goal is accomplished, the HCA can shift to its second goal, which is to understand how normal cell states transition into disease states^11^.

## Challenge of human cell-type classifications in the HCA

A typical scheme for assigning cell types to scRNA-seq data requires computational analysis and a workflow consisting of preprocessing and downstream analysis steps. Preprocessing is necessary to correct biological and technical noise caused by experimental errors and sample variabilities, thus contributing to quality control and normalization, whereas downstream analysis aims to cluster single-cell data on the basis of similarities in profiles and assign cell types using various computational tools depending on the data type^12,13^. Each cell type can be identified by a uniquely characteristic transcript pattern of gene expressions (i.e., RNA signature) or a uniquely characteristic transcript profile^14^. However, because the number of clusters depends on the algorithms and parameters, the results are not generally optimized for separating subtypes or states of the same cell type (e.g., cell cycle phase differences)^15^.

In this regard, several clustering approaches have been developed, including partition type (such as *k*-means and self-organizing maps), hierarchical clustering, graph-based (such as spectral clustering or clique detection), mixture model (such as Seurat), density-based, neural network type, ensemble, and affinity propagation type^16–18^. However, most of these approaches require parameter settings, and to complicate matters, some depend on random values. Consequently, the resulting clusters could vary in number and cluster members. Detailed reviews of these approaches can be found in recent papers^15,19^. In addition, a variety of software tools are available for clustering approaches, and the number is constantly growing.

Alternatively, the identification of a known cell type by its RNA signature can be performed using a cell−cell similarity search. In this case, researchers can identify known cell types and find similar or related unknown cell types by using hitherto unknown relationships. Software programs for cell−cell similarity search analyses include CellAtlasSearch^20^, CellSim^21^, and Cell BLAST^22^, all of which are tailored to different needs. For example, Cell BLAST has a “special tuning” mode for handling batch effects between a query and reference. CellSim calculates the similarity of different cells on the basis of cell ontology and molecular networks and has a feature that allows users to identify the cell type by entering a list of genes. Finally, CellAtlasSearch is tailored for handling ultra-large RNA-seq data through parallel screening of tens of thousands of single cells using efficient clustering methods. However, these methods are typically dependent on existing cell data and thus are effective for cell-type identification that demands large amounts of annotated cell-type entries.

Another approach for assigning cell types is the integration of gene expression information and spatial information of the individual cells to provide a molecular description of each cell type in the context of the tissue microenvironment. Recently, developed computational methods allow, in principle, the reconstruction of a spatial map of tissues using scRNA-seq data^23–25^. scRNA-seq-based maps have revealed cell-type-specific functions in the liver^26^, blastocyst^27^, and growth plate^28^. The expression of cell adhesion genes and specific gene functions defined by Gene Ontology (GO) terms is being used to develop new tools for single-cell 3D transcriptome analysis that enhance spatial prediction^29,30^. Although the identification of cell types in a spatial context is expected to yield more information relevant to the in vivo environment, these cutting-edge approaches are still at the elementary stage and need further improvement before they can be widely used.

To expedite methods for cell-type identification, the HCA project has multiple committees and working groups that coordinate the efforts of independent research groups and unify the results of scRNA-seq. Typically, the research groups perform all the data preparation, acquisition, and analysis, and the HCA committees provide the general framework, guidelines, and data repository space. The DCP team supplies vetted algorithms for data processing on the portal, and the research groups choose the algorithms for data processing, depositing the data into the HCA database under controlled pipelines. Hence, the cell-type classification, which is part of the RNA-seq data analysis, is performed separately by various groups, with the results processed by the cooperating DCP team according to HCA guidelines to ensure consistency in cell-type assignment and authentication.

## History of cell-type classification

With the advances in stem cell research that have made it possible to engineer cells for cell therapies and drug discovery, the identification and authentication of cell types have become emerging priorities in the biological community^31^. The greatest problem in the authentication of human cells lies in the lack of integrated standard metrics for cell morphology, gene expression, and molecular markers^32^. Considering that scRNA-seq data are usually confounded by a high degree of noise^33^ and that current scientific communities use highly variable methodologies for scRNA-seq data analysis^34^, it is very difficult to standardize the data and harmonize it with classical morphology and marker maps. Accordingly, the HCA project has invested both time and resources to study ways of handling technical and biological noise affecting data reproducibility and the degradation of biopsy samples^35^.

Historically, the characterization of cell types was based on histological, i.e., anatomical, morphological, and functional, criteria^36^. For example, the earliest attempt to classify cells in the nervous system was based on histological characteristics, such as the locations from which the cells were obtained, cell morphology, and the presence of certain molecular markers^37^. Since then, the location (i.e., cerebral cortex, cardiac muscle, or stomach), cell morphology (i.e., fibroblast-like or epithelial-like), and molecular markers (such as CD75-positive cells, etc.) have been accepted by the scientific community as the three main pillars for defining cell types^38–41^. To accelerate cell-type identification, several cell marker databases are available (Table 1).

**Table 1:** Online cell marker databases as of November 1, 2019.

Database	No. of markers	Cell type	Species	Characteristic	PMID	Address
Labome	226	7 major cell types and several others	Human	No distinction between diseased or healthy states. Few cell types are covered compared with other databases in this table. Source references are provided. No search engine. Only experimentally confirmed markers are included. This collection is most useful for searching verified markers.	N/A^42^	https://www.labome.com/method/Cell-Markers.html
CellFinder	553,905	3394 cell types and 50,951 cell lines	Human, mouse	Omics data are prioritized. In many cases, users must analyze raw data. No distinction between verified markers and ambiguous markers. This database is most useful for omics data exploration.	PMC3965082 ^43^	http://www.cellfinder.org/
CellMarker	22,753	467 human cell types and 389 mouse cell types	Human, mouse	A major portion of markers are for cancer cells. No distinction between experimentally confirmed markers and ones gathered through omics. No distinction between verified markers and ambiguous markers. References are provided. This database is recommended for a first search of information.	PMC6323899 ^44^	http://biocc.hrbmu.edu.cn/CellMarker/
PanglaoDB	8230	178 cell types	Human, mouse	Markers for healthy and diseased adult and embryonic cells. Source references are not provided for the markers. Contains single-cell sequencing data and automatically updates the list of papers on single-cell sequencing. Because of the relatively small number of cell types, this database is good for narrowing down the list of markers given by other databases.	PMC6450036 ^45^	https://panglaodb.se/markers.html
SHOGoiN	2594	740 cell types	Human	Markers for healthy human cells including embryonic cells. Source references are provided. No distinction between verified markers and ambiguous markers. This database is most useful for a quick search by cell type.	PMC3204613 ^81^	https://stemcellinformatics.org/cell_marker/literatures

Labome provides a list of 226 markers for epithelial, dendritic, glial, bone marrow, natural killer, and other cell types^42^. CellFinder was the first database website of molecular markers and now features information on 3394 cell types, 50,951 cell lines, and 553,905 protein expressions^43^. However, CellFinder data are diverse for species (mammals, fish, invertebrates, bacteria, viruses, plants, etc.), and the site includes other data, such as microscopic and anatomical images, whole-genome expression profiles from RNA-seq and microarrays, etc. In other words, it is not a cell marker database per se but a collection of various data of different cell types, which makes looking for markers relatively difficult compared to the search in other databases.

The first database compiled exclusively with markers of human and mouse cells was CellMarker^44^, which currently features 13,605 cell markers for 467 cell types in 158 human tissue and 9148 cell markers for 389 cell types in 81 mouse tissues. The gene expression data in CellMarker originate from scRNA-seq studies, experimental studies, and microarrays. At approximately the same time, PanglaoDB was published^45^, providing data on 8230 markers collected from human and mouse scRNA-seq experiments, along with other types of data. The markers are grouped by cell type (178 cell types, 4644 genes, and 29 tissues), and the cell types are subsequently grouped into 26 organs and 3 germ layers. A typical cell type has 28 (median) gene markers, but some, such as fibroblasts, have more than 100 markers. The major drawback of this database is that it lacks the source from which the marker information was obtained and information on how the marker was originally found and used, thus making information verification impossible.

## Limitations of the current methods of cell-type classification

Despite the abovementioned databases, the new era of single-cell sequencing, which has revealed that markers can be expressed in different cells or at varying levels when cells are cultured in vitro, demands the reconsideration of marker-based cell-type classification methods. For example, the identification of mesenchymal stromal cells (MSCs) and the results of fate mapping in vivo have been problematic^46^. In addition, many markers often correspond to several cell types rather than a unique one, which may be related to the fact that MSCs encompass a number of different tissue-specific progenitor or stem cells. In other words, some markers are of mixed cell types^47,48^. Moreover, the expression levels of some markers make it difficult to discriminate among cell types^49^. For example, mature monocytes are usually characterized by the expression of CD33, CD11b, CD14, HLA-DR, and CD16, whereas granulocytes are characterized by the expression of CD33, CD11b, CD15, and CD66b. However, CD15 is expressed at low levels on monocytes in some anti-CD15 clones. Conversely, in disease states, CD14 can be variably expressed in neutrophils^50^. Notably, current markers all share the common feature that they are expressed on the cell surface. Intracellular markers such as microRNA (miRNA) could enhance the specific marker profile of a given cell type^51^. The combination of surface and intracellular markers for cell-type classification has yet to be fully developed in any database.

**Fig. 1:**
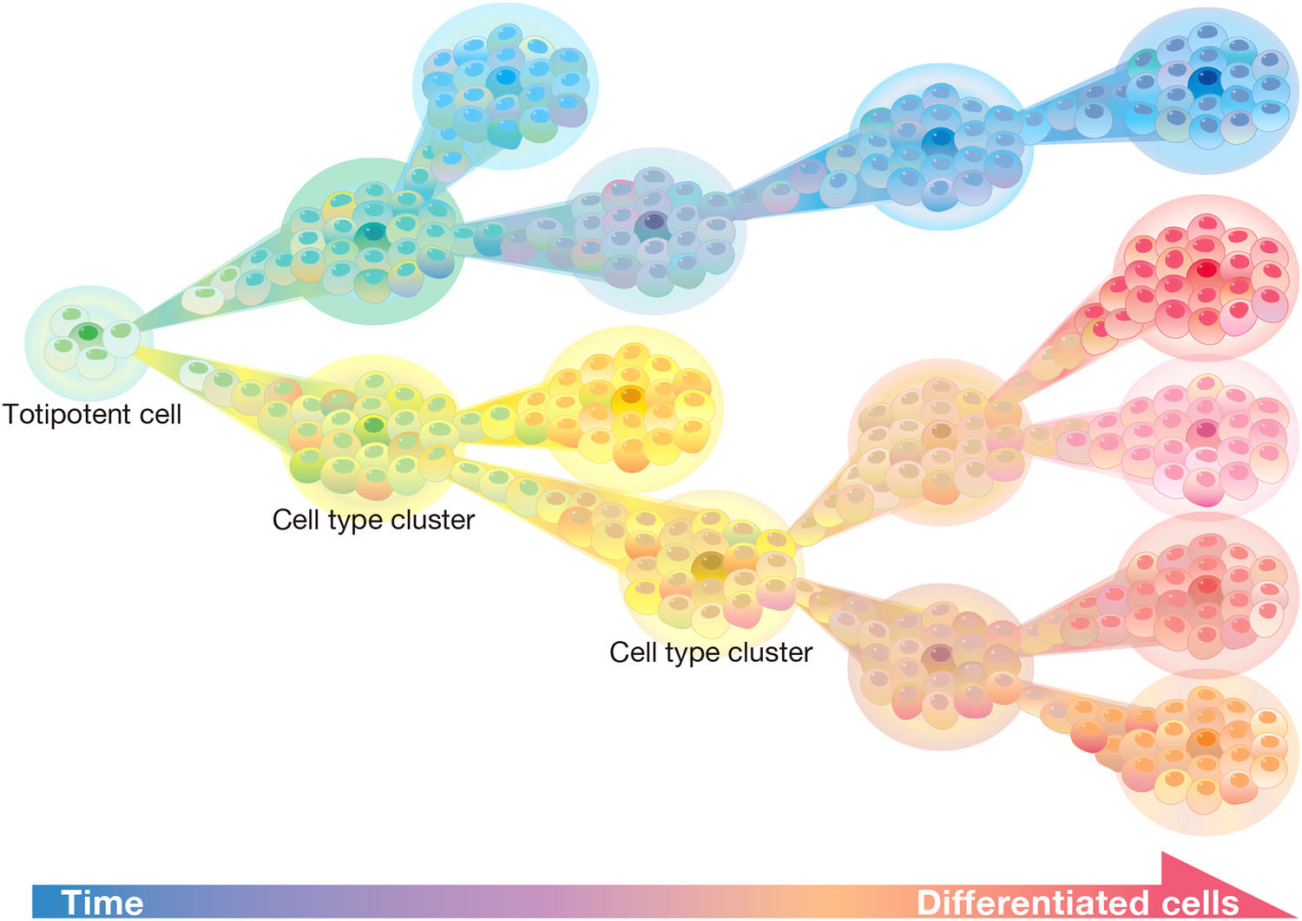
Renewed concept of cell types that takes into account the cell state continuum and diversity, as well as the state stability, of a cell type. Classically, the continuum was thought to be unidirectional, but cell reprogramming induces cells to regress to a previous state and/or take a different trajectory.

Another problem is the heterogeneity of cell states. In some cases, heterogeneity, such as that during states of immaturity or senescence, blurs discrete states, leaving a continuous spectrum that greatly complicates cell-type classification (Fig. 1). In such cases, cell types may seem to have hundreds of variants^52,53^. This lack of clarity is a major issue in clinical applications using cell therapies, for which cells are cultured in vitro to generate and stabilize a specific cell type. For example, for Parkinson’s disease, the differentiation of pluripotent stem cells (PSCs) into specific neural cell types for cell therapies demands extremely precise markers^54^. As with their use as intercellular markers, the application of miRNA would be helpful. Another important problem involves changes in cell behavior and cell composition under different conditions or even within the same culture. These changes compromise stable cell phenotypes across cell populations over the course of an experiment, leading to confusing experimental interpretations^55^.

## Emerging concepts for cell-type classification

Accordingly, several new concepts for cell-type authentication have been proposed. Evolutionary biologists have developed a classification on the basis of the evolution of gene expression states^56^. In this proposition, the cell type is defined by a set of changes in the “core regulatory complex” (CoRC) of transcription factors that regulate cell-type-specific traits. From this point of view, cell types are defined by evolutionary units that differ according to their evolutionary lineages rather than their phenotypic similarities and are characterized by their ability to evolve gene expression states independently of each other. Thus, a gene regulatory network that defines the cell type would include “master” transcription factors that control downstream effector genes^57–59^. A classic example is the transformation of fibroblasts into skeletal muscle cells by the forced expression of the myoblast determination protein (MYOD) transcription factor^60^. The discovery of induced pluripotent stem cells (iPSCs) cemented the notion that master transcription factors can determine cell type because, through reprogramming to iPSCs and their subsequent differentiation, nearly any cell type can be converted into any other cell type^61,62^. Later works in neuronal lineages^63^, the transcription factor competition observed in embryonic stem cells^64^, and pancreatic cell transdifferentiation^65^ support this idea.

Another evolutionary approach for cell-type classification is the construction of a hierarchy to describe the relationships between cells, which is analogous to how taxonomy hierarchies are created to describe the relationships between species^66^. Based on this approach, we proposed a “periodic table” for cell types^67^. This proposal aims to distinguish cell types from cell states, in which the periods and groups correspond to developmental trajectories and stages of differentiation^68^. scRNA-seq has paved the way for new interpretations of cell states. For example, in the epigenetic landscapes described by Waddington^69^, it was originally assumed that cell states follow continuous trajectories that branch at cell-fate decision points, but these decision points have since been refined into transition states^70^. Whereas Waddington assumed that the decision points were deterministic, the transition states in the modernized version are stochastic and the related signaling networks are probabilistic. This concept is meant to accommodate the gene expression heterogeneity encountered in real-world single-cell data. Indeed, the identification and characterization of cell transition states is one of the biggest challenges in single-cell transcriptomics^71^. Dimension reduction techniques, such as principal components analysis (PCA)^72^ and t-distributed stochastic neighbor embedding (tSNE)^73^, and graph and community detection algorithms, such as consensus clustering^74^, SNN-Cliq^75^, and Seurat^18^, have been developed and utilized to identify cell transition states. Fully comprehending the influence of cell transition states on cell types at the single-cell level will require both new tools and parameters^76^. Finally, although the results are still in the rudimentary stage, recent research has made automated cluster annotation available for unbiased cell-type identification. This type of annotation is rapid and allows investigators to forgo the manual clustering step by combining annotation and clustering. Numerous tools based on various algorithms have been developed to facilitate automated cell identification methods (for a detailed review, see Abdelaal et al.^77^).

## Proposing a data-driven cell-type definition

To help consolidate the many opinions about cell-type classification and provide data-related guidelines for cell-type authentication for clinical application, the International Cell Type Authentication Committee (ICTAC) was created (https://cell-type.org/). The ICTAC was launched to establish criteria and processes for defining, determining, and authenticating all human cell types. Its mission is to help scientific communities identify cell types and provide systematic information on cell classifications. The ICTAC originated from the International Stem Cell Banking Initiative (ISCBI) (https://www.iscbi.org/)^78^, an organization that focuses on practical issues in cell banking and regenerative medicine. More than 300 stem cell and policy professionals from 28 countries are part of the ISCBI community and are working together to advance stem cell research and biobanking along with developing regulations and public policy. The ISCBI is managed by an executive board, with delegates and steering group members of the community closely collaborating with the International Human Pluripotent Stem Cell Registry (hPSCreg)^79,80^. The first task of the ICTAC is to integrate existing cell databases, such as SHOGoiN^81^, CellFinder^43^, and Cell Ontology^82^, to comprehend information on existing cell-type definitions. One of the key functions of the ICTAC is to provide a tool that can process the massive amount of accumulating single-cell data to classify new cell types that do not fit into existing definitions or cannot be identified by cell-matching software, such as CellSim, CellAtlasSearch, or Cell BLAST.

The ICTAC proposed the concept of reference cell types (RCTs), which are defined by an integrative examination of core properties (species, physiological system, source age, and markers) and additional attributes (functions including potency, morphology, developmental origin, omics, and environmental conditions) and are constantly updated by experts of relevant tissues and organs. RCTs provide a framework for identifying and authenticating new and known cell types. Ultimately, RCTs are designed to support cell-type classifications in various communities, including stem cell banking initiatives and massive-scale single-cell sequencing projects.

## Cell type authentication for regenerative medicine

In the field of regenerative medicine, PSCs such as the aforementioned iPSCs have tremendous potential because of their capacity to differentiate into most cell types in the human body. Moreover, recent technological progress has made it possible to produce PSCs on a large scale^83^ and generate significant resources for a large number of well-characterized and documented PSCs (e.g., https://fujifilmcdi.com/the-cirm-ipsc-bank, https://ebisc.org^84^). However, in addition to scalability, the clinical translation of PSCs depends on fast and reliable ways to assess quality and safety in terms of three important properties: cellular identification, differentiation potency, and malignant potential^85^.

For example, the establishment of PSC-derived platelets as a substitute for primary donor cells can compensate for anticipated donor shortages and are useful against platelet transfusion refractoriness^86^. In this system, PSCs need to be differentiated into megakaryocytes, which are then cultured in bioreactors to shed platelets^87^. However, platelet production by iPSC-derived megakaryocytes is heterogeneous, and many biochemical and biophysical approaches have been attempted to enhance and homogenize their production^88^. Ultimately, the characterization of the best megakaryocytes is lacking. In other words, the existing classification of the megakaryocytes is too broad to identify the cells that are optimal for platelet production, thereby resulting in inefficient platelet production. This problem could be solved by identifying markers associated with platelet-producing megakaryocytes and developing differentiation protocols aimed at the selection of the relevant cell types.

Differentiation efficacy depends on PSC quality. There are several methods for ensuring high PSC quality that are based on assessing the potency of PSC differentiation into cells of the three germ layers. The gold standard is the teratoma assay^89,90^, in which the differentiation capacity of a PSC line in vivo is assessed by grafting the cells into immunodeficient mice. In addition to differentiation capacity, this assay enables the assessment of viability, histotypic organization, and carcinogenicity at the same time. However, the assay is lengthy and laborious, and it requires experts in pathological assessment and the use of experimental animals^91,92^. Furthermore, in a comparison of the methods used for and the results obtained from teratoma assays performed at 18 centers worldwide, the ISCBI found that both the test methods and test results varied substantially among expert centers^93^. Another quality check method is the embryoid body (EB) assay, which enables the monitoring of differentiation capacity in vitro. EBs are cell aggregates that spontaneously differentiate into three developmental germ layers when cultured in suspension^94^. This approach is more standardized than the teratoma assay and considered more robust by some researchers^95^. Contributing to its favorability, an EB assay can be combined relatively easily with a bioinformatics analysis of gene expression profiles^96–98^.

Finally, the detection of pluripotency-specific markers, such as alkaline phosphatase^99^, Nanog, and Oct4, as well as other mRNAs and proteins, is another way to perform a quality check of PSC characteristics. The detection of these markers is usually performed using flow cytometry, RT-qPCR, and cell-staining techniques^100^. A number of markers can be used to identify PSC types, such as the naive PSC state or the high-yield expansion PSC state^101–104^, and to measure the quality of the PSCs^105^. At the single-cell level, pluripotency can be assessed with scRNA-seq and bioinformatics tools. For example, a PluriTest® assay is used to distinguish typical PSCs from other PSC-like populations through machine learning that is based on the transcriptomes of ideal cell lines and control PSC lines^106^. However, functional differentiation assays are still required to exclude false-positive results. Other tools, such as SLICE^107^, SCENT^108^, and Epi-Pluri-Score^109^, allow researchers to quantify cell potency and cellular differentiation by entropy analysis. However, a recent multinational study of a range of pluripotency assays concluded that the demonstration of at least some capacity for in vitro or in vivo differentiation was important for the veracity of the results^85^.

## Guidelines for big data generation, storage, and management

As described above, numerous groups are working internationally to generate human omics data not only for tissues and bulk cells but also for millions of individual cells. The gathered data have the potential to greatly advance experimentation practices and quality checks for cells intended for clinical applications. However, for this goal to be realized, the data must be structured, and new algorithms that can efficiently curate the data are needed. Accordingly, members of the ISCBI have proposed Minimum Information About a Cellular Assay for Regenerative Medicine (MIACARM) guidelines, which are directed first and foremost at stem cell banks, although MIACARM can also be used to structure data formats of cellular assays in general^110^. MIACARM is based on the Minimum Information About a Cellular Assay (MIACA), the first attempt at creating a reporting format for describing functional research on cell lines^111^. Unlike MIACA, MIACARM sets guidelines for human cells used in medical applications or single-cell analysis, including omics. The proposed guidelines have the potential to enhance information flow in stem cell research that aims to produce clinical-grade cells for therapies. As an extension of MIACARM, which currently targets source cell characterization (MIACARM-I) and stem cell characterization (MIACARM-II), the ISCBI community is engaged in plans to provide guidelines for characterizing differentiated cells (MIACARM-III) using ICTAC proposals for cell authentication based on the framework developed for RCTs.

## Conclusion

It is obvious that the body constitutes a myriad of different cell types that have specific functions. Advances in microscopy, histology, and, now, omics technologies have made it clear that the list of cell types is much longer than originally imagined and that our understanding of molecular expressions in systems both in vitro and in vivo is still at the nascent stage. Moreover, the concept of cell reprogramming has taught us that cells can be in constant flux, oscillating between cell types. The ability to define these different cell types is crucial for understanding natural development, including the development of diseased states, and for producing cells for clinical therapies. Critical points are a consensus definition of cell types and the data required for their authentication. In addition, it is vital to ensure accurate and traceable links between precious resources of biological materials and the associated data sets to make full use of both biological and electronic resources and promote reproducibility in scientific data. The many existing databases and the massive data already accumulated affirm the need for the scientific community to work together in creating a universal standard.
